# Evaluating gut microbiota profiles from archived fecal samples

**DOI:** 10.1186/s12876-018-0896-6

**Published:** 2018-11-08

**Authors:** Trine B. Rounge, Roger Meisal, Jan Inge Nordby, Ole Herman Ambur, Thomas de Lange, Geir Hoff

**Affiliations:** 1 Department of Research, Cancer Registry of Norway, Oslo, Norway; 2 Department of Microbiology and Infection Control, Akershus University Hospital, Lørenskog, Norway; 3 Department of Medical Biochemistry, Oslo University Hospital, Oslo, Norway; 4 Department of Life Sciences and Health, OsloMet – Oslo Metropolitan University, Oslo, Norway; 5 Section for Bowel Cancer Screening, Cancer Registry of Norway, Oslo, Norway; 6 Department of Research and Development, Telemark Hospital, Skien, Norway; 7 Institute of Clinical Medicine, University of Oslo, Oslo, Norway

**Keywords:** Fecal samples, Microbiota, Archived samples, Fecal immunochemical tests, Storage, Diversity

## Abstract

**Background:**

Associations between colorectal cancer and microbiota have been identified. Archived fecal samples might be valuable sample sources for investigating causality in carcinogenesis and biomarkers discovery due to the potential of performing longitudinal studies. However, the quality, quantity and stability of the gut microbiota in these fecal samples must be assessed prior to such studies. We evaluated i) cross-contamination during analysis for fecal blood and ii) evaporation in stored perforated fecal immunochemical tests (iFOBT) samples, iii) temperature stability as well as iv) comparison of the gut microbiota diversity and composition in archived, iFOBT and fresh fecal samples in order to assess feasibility of large scale microbiota studies.

**Methods:**

The microbiota profiles were obtained by sequencing the V3-V4 region of 16S rDNA gene.

**Results:**

The iFOBT does not introduce any cross-sample contamination detectable by qPCR. Neither could we detect evaporation during freeze-thaw cycle of perforated iFOBT samples. Our results confirm room temperature stability of the gut microbiome. Diverse microbial profiles were achieved in 100% of fresh, 81% of long-term archived and 96% of iFOBT samples. Microbial diversity and composition were comparable between fresh and iFOBT samples, however, diversity differed significantly between long-term archived, fresh and iFOBT samples.

**Conclusion:**

Our data showed that it is feasible to exploit archived fecal sample sets originally collected for testing of fecal blood. The advantages of using these sample sets for microbial biomarker discovery and longitudinal observational studies are the availability of high-quality diagnostic and follow-up data. However, care must be taken when microbiota are profiled in long-term archived fecal samples.

**Electronic supplementary material:**

The online version of this article (10.1186/s12876-018-0896-6) contains supplementary material, which is available to authorized users.

## Background

Colorectal cancer (CRC) is the third most frequently diagnosed cancer with more than 1.4 million new cases diagnosed annually worldwide [1, 2]. The World Health Organization and the European Union recommend CRC screening to detect CRC and its precursors at a curable stage. However, the most frequently used non-invasive fecal tests (guaiac-based fecal occult blood test (gFOBT) or an immunochemical FOBT (iFOBT) have inadequate sensitivity [1, 3]. A multi target stool DNA test combined with iFOBT has shown high sensitivity, but poor specificity [3]. Highly specific and sensitive non-invasive screening tests are urgently needed. Thus, identifying fecal biomarkers for CRC risk assessment, early detection and prognosis is a priority.

Cross-sectional human studies have shown associations between bacteria and colorectal cancer [4, 5] and the microbiota differs between neoplastic lesions and healthy mucosa [6, 7]. The gut microbiome is less studied in the early stages of carcinogenesis. Hale et al. [8], showed that the bile-tolerant microbes *Bilophila, Desulfovibrio*, proinflammatory bacteria in the genus *Mogibacterium*, and multiple *Bacteroidetes* species are more abundant in adenoma cases. A microbiome biomarker based on 16S sequencing combined with FOBT screening was shown to increase CRC detection compared to FOBT screening [9]. However, the studies lack co-variables and could only marginally improve detection of adenomas [9, 10]. It is probable that, with further development, the gut microbiome can be used to detect the presence of precancerous and cancerous lesions [11]. However, human studies have been limited to cross-sectional case-control series showing bacterial associations to CRC rather than a causative link to future risk which requires a prospective, observational study design [12, 13].

Fecal samples from CRC screening can provide large number of samples with diagnostic data needed for biomarker discovery in longitudinal studies [14]. Long-term stored biobanks, collected before preservatives were common, may have follow-up data from long-term projects or national registries, such as the NORCCAP cohort [15, 16]. Biobanks of FOBT samples are often large and collected at all stages of CRC development, such as the The Bowel Cancer Screening in Norway (BCSN trial) [17]. These biobanks are invaluable for assessing the temporal dynamics of the microbiota-CRC relationship from years before diagnosis to times after treatment.

Nonetheless, using fecal samples not originally intended for microbial profiling may introduce technical challenges due to inadequate material and varying sample handling and storage. Technical aspects regarding storage conditions, freeze-thaw effects and use of preservative media have previously been studied in fresh fecal samples [14, 18–21]. Good microbiota concordance has been shown between fresh stool and FOBT cards [22]. However, all aspects of how sample processing and storage may potentially influence the microbiota need to be investigated.

The aims of this study were therefore to investigate the feasibility of using iFOBT and long-term archived fecal samples frozen for up to 16 years for 16S rDNA sequencing. Specifically, cross-contamination during the test for fecal blood, reduction of sample quality due to storage and handling of perforated iFOBT samples, temperature stability and comparison of the gut microbiota diversity and composition was evaluated.

## Methods

### Samples and design

The BCSN trial is a comparative effectiveness research trial randomizing 140.000 persons aged 50–74 years to be screened for CRC either with flexible sigmoidoscopy (FS) or iFOBT [17]. About 70.000 individuals have been invited for iFOBT screening and will deliver iFOBT samples every other year for 10 years. The sampling kits for iFOBT are mailed to the participants and collected by return mail with a shipping time of 2–6 days. The stool samples are collected on plastic sticks designed to catch about 10 μg stool and then stored in 2 ml buffer containing HEPES buffer (4-(2-hydroxyethyl)-1-piperazineethanesulfonic acid), BSA (Bovine serum albumin) and sodium azide. The participants were asked to mail the sample to the lab as soon as possible. On the day of arrival to the laboratory, the samples were analyzed with an immunochemical test for human blood (globin) (Eiken Chemicals Ltd., Tokyo, Japan) and the perforated iFOBT tube was frozen at − 80 **°**C immediately thereafter. We randomly selected 50 anonymized iFOBT samples among the participants in the BCSN trial for this study. The samples have not previously been thawed.

The NORCCAP study was a randomized flexible sigmoidoscopy screening clinical trial of 100,210 individuals aged 50 to 64 years from the populations in two Norwegian counties (Oslo and Telemark). The trial was performed during 1999–2000 (55–64–year age group) and in 2001 (50–54–year age group) [15, 16]. The intervention group received flexible sigmoidoscopy and delivered stool samples for iFOBT. To-date the trial has about 4800 stool samples stored at − 30 **°**C. 50 feces samples randomly selected from NORCCAP participants with normal sigmoidoscopy were included in this study. Stools were sampled in 20 ml vials by the screening participants at home and kept for 1–7 days in their home freeze (− 20 **°**C) before delivery to the screening centre when attending FS screening. Samples were transported in the 20 ml vials with an outer transport tube. Most participants had less than 30 min travel to the screening centre, but partial thawing during this transport is likely. At the screening centers, stools were stored at − 20 **°**C for up to 1 week before transport for up to 2 h in insulated transport boxes to a central − 30 **°**C storage room with alarm systems. Some samples may have been partially thawed to provide material for non-microbial research purposes.

Fresh samples from eight anonymous, presumably healthy individuals, four men and four women, aged 50 to 64 years were collected in 2015. Each individual froze one part of their stool sample directly and one part was stored at room temperature for 48 h before it was frozen at home. Partial thawing may have occurred during transport to the lab. All samples were frozen at − 20 **°**C in the lab until DNA extraction.

The collection of iFOBT and archived samples was approved by the Regional Committees for Medical and Health Research Ethics in South-Eastern Norway (2011/1272 and 2010/3087 A, respectively). An additional ethical approval for the feasibility study was not required since all samples were anonymized and the purpose of the study was method related, as stated by the Norwegian regional ethics committee (Regional Committees for Medical and Health Research Ethics in South-Eastern Norway, ref. 2015/9).

### Cross-contamination during iFOBT analysis

In the automatized analysis of iFOBT, the same instrument needle extracts liquid from all samples in a consecutive order with intermediary washing. To investigate if this may result in cross-contamination of the samples, a test sample series alternating between 200 ng/μl human DNA (Roche #11691112001) and water was prepared. This sample series was run on the desktop instrument OC-Sensor Diana (Eiken Chemicals,Tokyo, Japan). A qPCR measurement of human DNA beta-globin in the 40 water samples and two positive controls were analyzed in two replicates in 25 μl total TaqMan universal PCR master mix (Thermofisher Scientific, Waltham, USA) reactions using 5 μl template [23].

### Sample handling and storage effects on perforated iFOBT samples

The iFOBT tubes are perforated during iFOBT analysis. Evaporation during the freezing process, sublimation during storage or condensation during thawing of these “open” tubes might increase or decrease sample volume indicating that contamination of samples due to these processes might be possible. To asses if samples quality could be diminished by such processes we tested for changes in sample volume by freezing 50 perforated iFOBT tubes with and without parafilm and parafilm with rubber bands. We compared the weight of the 50 tubes before and after 6 months in the freezer (− 80 °C), 3 h after thawing.

### DNA isolation, PCR and sequencing

All samples were processed in Lysing Matrix E tubes with silica beads. Sample input was about 10 μl, approximately 10 mg, for the solid archived fecal samples and 500 μl for the iFOBT samples. Dry weight of feces varies in the iFOBT samples, however, we estimate that about 2.5–5 μg feces were used in the protocol. PBS (phosphate-buffered saline) buffer was added to a total liquid volume of 1.1 ml prior to mechanical lysis on a FastPrep 24 and centrifugation at 400×g for 1 min. Nucleic acids were extracted on an automated platform, QIAsymphony, using a standard nucleic acid extraction protocol that included pretreatment with ProteinaseK and a final elution in 85 μl. To account for the level of microbiological contamination from the environment and reagents an extraction-negative control (ENC) containing all the reagents except for sample material was processed in each batch of samples. Two additional negative control samples were included in the PCR, one with water and one with 10 ng/μl human DNA. Due to experimentally encountered inhibition of PCR in the archived samples, these were diluted 1:5 before used as template for PCR. All other samples were used directly in PCR without dilution.

A two-step PCR protocol was used. The primers S-D-Bact-0341-b-S-17 and S-D-Bact-0785-a-A-21 [24] were first used to amplify a 16S rDNA in the V3-V4 region, to produce a 464 nucleotide (nt) long amplicon. PCR was performed in 20 μl reaction volumes with 1× Phusion Master Mix, 0.25 μM each 16S primer and 2 μl template under the following conditions; initial denaturation 98 °C for 30 s and then 27 cycles of 98 °C for 10 s, 62 °C for 30 s and 72 °C for 15 s, before a final extension at 72 °C for 10 min and storage at 10 °C. PCR cleanup was done using partly modified PERFORMA DTR V3 96-well Short Plates and QuickStep™2, 96 Well PCR Purification kit protocols. All samples PCR products were tested on 1% agarose gels using 5 μl sample. Then in the second round of PCR, indexes were added. The index PCR was also performed in 20 μl volumes using 1× Phusion Master Mix, 0.5 μM each index primer, and 1 μl of template DNA from the previous PCR, diluted 1:100.

Sequencing of the amplicons was performed on an Illumina MiSeq (Illumina) desktop sequencer using V3 chemistry 2 × 300 cycle kits. This protocol has shown high reproducibility and repeatability and very little contamination [25].

### Bioinformatics and statistics

Sequencing quality filtering and processing were carried out using the MiSeq SOP and the Mothur pipeline (v.1.36.1) [26, 27]. To ensure high-quality data for analysis, sequence reads containing ambiguous bases, homopolymers > 8 bp, more than one mismatch in the primer sequence, or sequences under the default per base quality score were removed. Assembled reads > 460 bp in length, singletons and chimeric sequences identified with the UCHIME algorithm [27] were excluded from the analyses. The high-quality assembled sequences (contigs) were aligned to the Silva 16S rRNA database (v.119) [28] and clustered into operational taxonomic units (OTUs) at a cut-off value > 98% in a closed reference OTU approach. OTUs were calculated at distance 0.02, and alpha diversity (Shannon index and inverse Simpson index) was calculated per sample. ﻿The number of assembled sequences for each sample was rarefied to 2000 to minimize the impacts of uneven sampling. Inverse Simpson’s index is weighted on dominant species, whereas the Shannon index assumes all species are represented [29]. To identify differences in species diversity between the groups, a two-way ANOVA and post hoc comparisons (Tukey Honest test of Significant Differences - TukeyHSD) of the observed number of OTUs, Inverse Simpson diversity and the Shannon index were used. Beta diversity (i.e., the variation in community composition between microbiota samples) was calculated between the sample types using the Bray–Curtis dissimilarity index [30]. Dissimilarities between sample groups were tested using ﻿permutational analysis of variance (PERMANOVA) implemented in the vegan (v. 2.5–2) R package and the “adonis” function. Differentially abundant OTUs were identified using the zero-inflated Gaussian model (fitZig), implemented in the metagenomeSeq package [31, 32]. The community composition data was transformed using the “decostand” function and divided by the margin total. Principal components analyses were done using the prcomp function in R. Heatmaps of the log transformed OTUs (for OTUs with more than 10 in counts) were produced using hierarchical clustering and Euclidean distance. To improve visualization only OTUs with a log sum > 20 for all samples, and > 2 for the fresh samples were shown. The clustering of these heatmaps were indistinguishable from heatmap with all OTUs included.

All of the ENC and negative controls, but also four iFOBTs and 14 archived samples did not produce sufficient aligned reads to calculate diversity.

## Results

### No cross-contamination during iFOBT or change in volume in perforated samples during storage

Potential transfer of DNA from sample to sample during iFOBT analysis was tested with qPCR. 79 of the 80 qPCR measurements were negative. i.e. no contamination. One sample had a cycle threshold (Ct) value of 40.7, also considered a negative result. The positive controls were positive on the qPCR (Ct values < 30).

The weight difference of 50 perforated iFOBT tubes before freezing (383.6 g) and after 6 months in the freezer (383.7 g) was 0.1 g or 0.03%. The weight difference of 50 perforated iFOBT tubes packed in parafilm to prevent change in volume before freezing (384,5 g) and after freezing (384,9 g) was 0.4 g or 0.1%. Some condensation on the tube surface was observed.

### Microbiota differs in iFOBT, fresh and archived samples

**Fig. 1:**
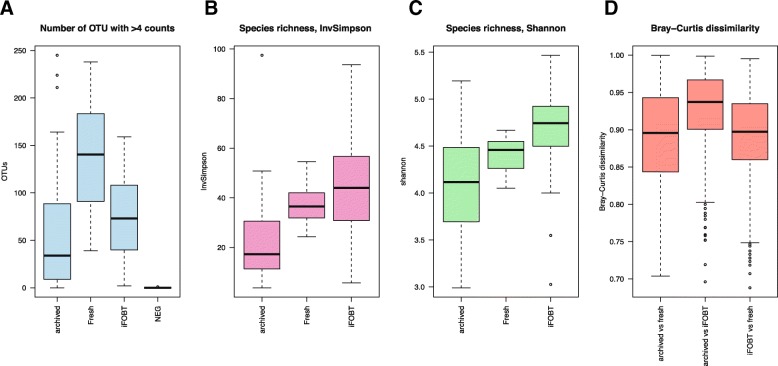
Alpha and beta diversity in iFOBT, fresh and archived samples. **a** Shows a boxplot of the number of observed OTUs in each sample group. **b** and **c** Shows boxplots of the Inverse Simpson index (**b**) and Shannon (**c**) index in fecal immunochemical tests (iFOBT samples, fresh fecal samples and fecal samples archived for approximately 16 years. The indexes are based on rarefied OTU data to minimize the impacts of uneven sampling. The Bray-Curtis dissimilarity index for comparisons of groups are shown (**d**)

In total, 4.2 million 16S rDNA contigs were assembled from the sequencing data. The fresh samples were sequenced separately, with a larger fraction of the sequencing pool per sample than the other sample groups, thus producing more reads, contigs and OTUs (Fig. 1a, Additional files 1, 2 and 3). Archived samples produced less OTUs than fresh and iFOBT samples (Table 1). Gel electrophoresis of the 16S rDNA PCR product from the archived fecal samples showed increased PCR efficiency when the DNA solutions were diluted 5-fold compared to undiluted DNA, signifying PCR inhibition. There were no signs of PCR inhibition in iFOBT samples.

**Table 1:** Overview of operational taxonomical unit (OTU) counts in each sample type

Sample groups	No. of samples	Mean no. contigs	Min no. contigs	Max no. contigs	Mean OTUs > 9 counts	Mean OTUs > 4 counts	Mean OTUs > 0 counts	Samples > 200 OTUs in total (%)	Samples > 500 OTUs in total (%)
iFOBT	49	84,861	5788	386,376	43	72	515	47 (96%)	46 (94%)
Archived (NORCCAP)	52	64,830	238	925,009	34	56	483	42 (81%)	36 (69%)
Fresh	8	314,252	87,303	751,297	109	158	770	8 (100%)	8 (100%)
Fresh (room temp 48 h)	8	286,571	56,485	646,383	79	120	547	8 (100%)	8 (100%)
ENC	6	151	21	2129	0	0	8	0 (0%)	0 (0%)
Negative controls	12	395	1	4486	0	0	10	0 (0%)	0 (0%)

### Diversity

The mean, maximum and minimum numbers of contigs (assembled reads) assigned to OTUs per sample group are shown in Table 1. The ENC and negative control samples produced very few OTUs. 96% of the iFOBT, 81% of the archived and 100% of the fresh samples passed a minimum relative abundance of 200 OTUs. The minimum relative OTU abundance chosen as a threshold was based on the sequencing depth and distribution of OTU counts (Additional file 1).

**Fig. 2:**
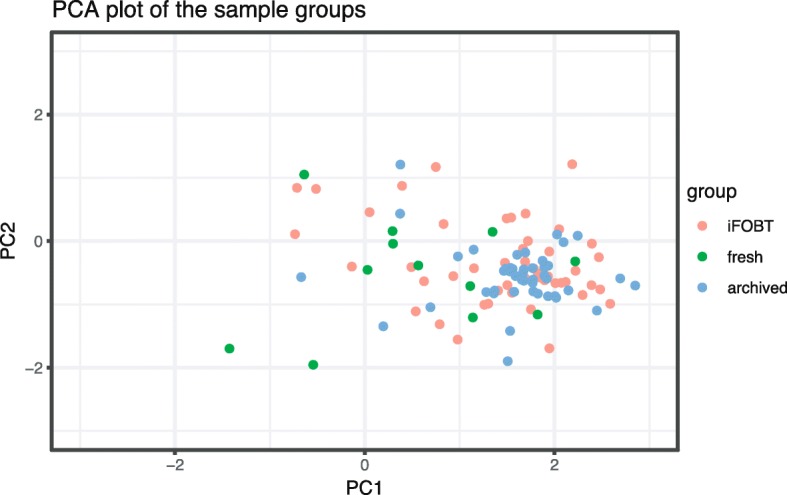
Principal component plot of the community composition of iFOBT, archived and fresh sample. The first and second principal components of the community composition of iFOBT samples from a screening trial in Norway (BCSN), archived samples from the NORCCAP cohort stored for about 16 years and fresh samples

We compared the diversity in iFOBT, fresh and archived samples using observed OTUs, and the Inverse Simpson and Shannon indexes from rarefied OTU tables (Fig. 1a, b, c and Additional file 4). Fresh and iFOBT samples were similar in mean diversity while the archived samples had statistically significant less diversity compared to iFOBT (*P*-value = 0.027 for Inverse Simpson and P-value = 0.06 for Shannon) and fresh (P-value < 0.001 for both Inverse Simpson and Shannon) in the ANOVA and TukeyHSD test. The Bray-Curtis dissimilarity index showed that the microbiota in iFOBT, archived and fresh samples differ (Fig. 1d and Additional file 5) and the PERMANOVA analysis showed that this difference is significant (*P*-value < 0.001, F = 1.4587). The first and second principal components showed overlapping community composition (Fig. 2).

### Relative abundance

**Fig. 3:**
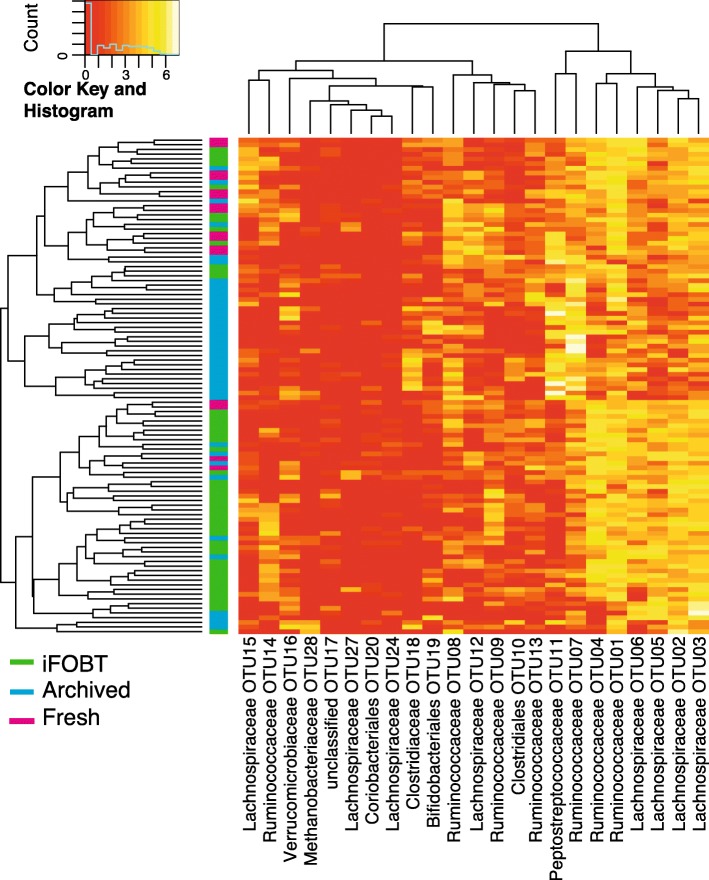
Clustering of archived, fresh and fecal immunochemical tests (iFOBT) fecal samples. Heatmap of the log transformed OTU table for all samples produced by hierarchical clustering and Euclidean distance. Only OTUs with a log sum > 20 were illustrated. iFOBT samples are marked in green, archived fecal samples stored for 14 to 16 years at − 30 °C are marked in blue and fresh samples are marked in pink. All samples are from presumably healthy individuals

The microbiota profiles differed between fresh and iFOBT vs. archived fecal samples (Fig. 3). More than 62% of the archived fecal samples clustered together, while the fresh and iFOBT samples were intermixed (Fig. 3). In particular, the OTUs 1 to 6 (rightmost vertical clade), including *Lachnospiraceae* and *Ruminococcaceae* family OTUs, were of high abundance in fresh and iFOBT samples, whereas OTU 7 and 11, representing the *Peptostreptococcaceae* and *Ruminococcaceae* family, were abundant in archived samples.

The zero-inflated log-normal mixture model identified 11 OTUs that significantly (*P*-value < 0.05) differed between archived and iFOBT samples (Table 2 and Additional file 6). The OTUs of the *Ruminococcaceae* family (OTU4, 14 and 9) and OTU5 *Lachnospiraceae* were significantly less abundant in archived samples. *Peptostreptococcaceae* (OTU11) and *Clostridiaceae* (OTU18) were more abundant in archived fecal samples.

**Table 2:** Differences in OTU abundance in archived and iFOBT samples

Family (order)	OTU	Log_2_ fold change	Standard error	*P*-values	Adj. P-values
Ruminococcaceae	OTU04	−1.232	0.201	8.23 × 10^− 10^	2.55 × 10^− 8^
Peptostreptococcaceae	OTU11	1.729	0.296	5.08 × 10^− 9^	7.87 × 10^− 8^
Clostridiaceae	OTU18	1.853	0.370	5.57 × 10^−7^	5.76 × 10^− 6^
Ruminococcaceae	OTU14	−1.526	0.333	4.64 × 10^− 6^	3.59 × 10^− 5^
Lachnospiraceae	OTU05	−0.816	0.197	3.31 × 10^− 5^	2.05 × 10^− 4^
Ruminococcaceae	OTU09	−0.755	0.273	5.68 × 10^− 3^	2.52 × 10^− 2^
Verrucomicrobiaceae	OTU16	0.846	0.306	5.69 × 10^− 3^	2.52 × 10^− 2^
Family_XIII_Incertae_Sedis	OTU21	0.980	0.372	8.37 × 10^− 3^	3.24 × 10^− 2^
Unclassified	OTU17	−0.869	0.340	1.06 × 10^− 2^	3.67 × 10^− 2^
Lachnospiraceae	OTU12	0.638	0.256	1.26 × 10^− 2^	3.92 × 10^− 2^
Bifidobacteriales	OTU19	0.730	0.303	1.58 × 10^− 2^	4.45 × 10^− 2^

### Stability of fecal microbial composition in room temperature

**Fig. 4:**
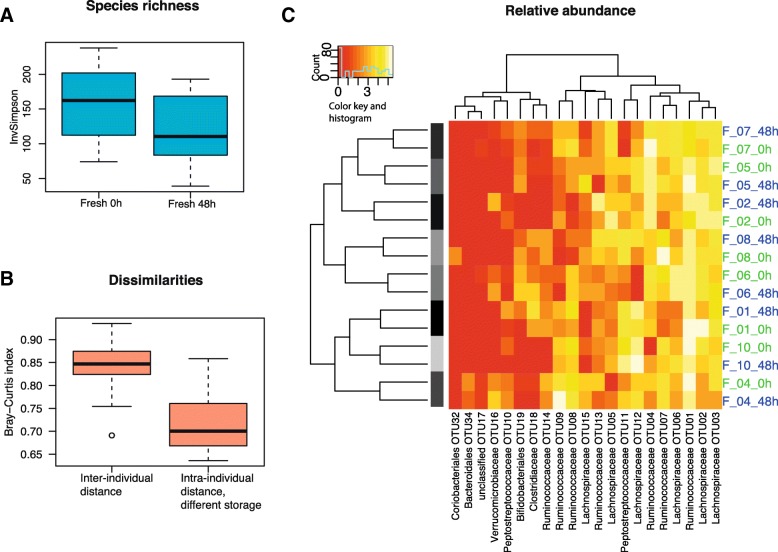
Microbiota profiles in fresh samples frozen directly and frozen after 48 h in room temperature. **a** Boxplot of Inverse Simpson alpha-diversity index in samples frozen directly and frozen after 48 h. **b** Bray-Curtis dissimilarities between samples from the same individuals with different storage conditions, and between different individuals regardless of room temperature storage. **c** Heatmap of the log transformed OTU table from paired fecal samples from 8 presumably healthy individuals (serial number 01 to 10). One part of the samples was directly frozen (0 h, marked with green text) and the other part of the samples was frozen after 48 h in room temperature (48 h, marked with blue text). Hierarchical clustering and Euclidean distance produced the clustering showing higher inter-person variability than intra-person variability. Only OTUs with a log sum > 2 were illustrated

The mean alpha-diversity did not differ significantly between samples that had been directly frozen and samples that were frozen after 48 h in room temperature (Observed OTU count P-value = 0.18, Inverse Simpson P-value = 0.65 and Shannon P-value = 0.94) (Fig. 4a and Additional file 4). Dissimilarities (Bray-Curtis) between sample pairs from the same individual with different room temperature storage time, was lower than between samples from different individuals (Fig. 4b). Unsupervised clustering of the log-transformed OTU relative abundance supports similarities between sample pairs (Fig. 4c). The log-normal mixture model identified no significant differences in relative abundance (Additional file 6) between samples stored in room temperature for 48 h and samples directly frozen (Additional file 7).

## Discussion

Fecal samples collected as part of CRC screening are usually intended for analyses of occult blood and not for studying the microbiota. Thus, it is necessary to assess the microbial DNA quantity, quality and biases introduced by sample collection and storage before these samples can be used in microbial epidemiological studies.

Our study has shown that it is feasible to profile the microbiota directly from iFOBT samples, also shown by others [9, 33]. Fecal samples archived for up to 16 years, collected as part of the NORCCAP study [17, 22], also produced microbial profiles sufficient for identification of inter-individual differences and association studies, although further improvement of the protocol such as homogenization of the samples [34], is required.

Microbial profiling with highly sensitive methods such as Next-Generation Sequencing will likely detect potential cross-sample contamination that may have occurred during analysis for blood in the iFOBT samples. This type of cross-contamination would distort the microbial profiles and make the samples unsuitable for epidemiological studies. However, our experiment using qPCR tests on runs with alternating blanks and concentrated DNA added to the iFOBT samples could not detect any cross-sample contamination. Our simple freezing tests indicated that storage of perforated iFOBT tubes will not change the volume of the samples and therefore likely not reduce the microbiota quality. We therefore believe that the iFOBT samples are eligible for analysis using sensitive methodologies. These results are also relevant for other tests, such as DNA methylation detection combined with testing for occult blood from the same collection device [35].

Proficient microbial profiling depends on complete lysis, here ensured by the use of both mechanical and enzymatic steps during the DNA isolation, primers that amplify as wide a target as possible (evaluated by Klindworth et al. [24]) and effective amplification. Bile salts and complex polysaccharides in feces [36, 37] and heme in blood [38] are substances that inhibit the PCR and reduce the amplification efficiency. Reducing these components in the PCRs with fecal DNA extracts will likely increase OTU counts and potentially the diversity in the archived fecal samples.

Our data show differences in microbial composition between the fresh, iFOBT and archived fecal samples. Species diversity in fresh samples is more similar to the iFOBT samples than to the long-term archived samples. OTU count differences are strongly related to sequencing depth in a non-saturated setting. Taking this into account, the observed OTUs are comparable between iFOBT and archived samples. The community compositions of these sample groups are overlapping, although there are significant differences in beta-diversity. RNAlater**®** has been shown to preserve the microbial components in fecal samples [19]. It is likely that the iFOBT buffer containing HEPES, BSA and sodium azide may also preserve the microbiota. The sodium azide should stop any bacterial growth as it is a strong anti-microbial agent.

To our knowledge, there are no studies of the stability of the microbiota in long-term stored archived samples. We showed significant reduction of species diversity in samples archived for up to 16 years. However, long-term stored samples have successfully been used to identify associations in fecal microbiota between colorectal cancer cases and controls [33, 39]. Samples used in these studies were freeze-dried and the lyophilates were pooled, mixed, and stored at − 40 °C. Large cohorts with follow up data and fecal samples that are processed in this manner are very rare to come by. Reduced alpha and beta diversity estimates have previously been shown for samples collected in no buffer compared to iFOBT samples and attributed to the thawing process [19]. Storage in domestic freezers, fluctuating in temperatures, has been shown to shift the abundances of the major taxa [34]. Thus, freeze-thaw cycles or difference in storage time in frost-free domestic freezers regardless of the long-term storage time might explain the difference in species diversity.

Differences in bacterial relative abundance were also observed between iFOBT and archived samples in our study. Particularly the common gut *Ruminococcaceae* family within the *Clostridia* order was less abundant in archived samples. The relative abundance of *Ruminococcus* species has also been shown to decline with storage time in samples from different persons [21]. Therefore, a study with mixed sample types may detect mostly sample type differences and mask true associations. Although, on their own and with an improved protocol, archived samples are of high value, particularly due to the possibility of doing longitudinal studies. These observed differences emphasize the need to analyze the iFOBT and the archived NORCCAP materials separately in future studies on prevalent and incidents CRC during years of follow-up.

Several studies have investigated the stability of microbiota in fecal samples stored for different length of time in room temperature using different study design, sample conservation, limitations and endpoint, with conflicting results. Most studies have reported small or insignificant changes of the microbial composition or diversity for feces samples stored in preservative solutions [18, 20, 21, 33, 34, 38, 40–42]. A comprehensive test of preservation methods showed that variability in microbiota was only minimally explained by preservation medium and that storage effects are small compared to individual differences [42]. Fecal samples without preservative, comparable to our long-term archived samples, stored in freezers for 8 weeks, showed lower diversity than between individuals [42].

Flores et al. [19] have showed significant changes between replicates for rare OTUs (< 1% abundance), but otherwise stable microbiota for feces stored in RNAlater®. Beta diversity was stable for samples both with and without preservative. Contrary to our results, an increased variance for freezing delays was shown [19]. Two other studies have reported effect of storage on microbial composition and diversity [43, 44]. There is a need for longitudinal studies subjecting aliquots of the same samples to different storage for different length of time over years.

Ahn and co-workers [39] identified large differences in relative microbial abundance with reported odd ratios in the range of 4 to 5 between colorectal cancer cases and controls, reproduced by Vogtmann et al. [33]. More studies are needed in order to determine if the effects of delayed freezing and preservation media may distort the associative signals from for example colorectal cancer - control studies.

Although limited by samples stored in a buffer designed for iFOBT analyses, potential exposure to thawing during transport, use of home freezers and storage without preservative, our results indicate that samples collected during screening, not initially intended for the purpose, may be used for microbial profiling. The disadvantages of using screening samples are variable quality of material and insufficient control of sampling, storage and freeze-thaw cycles. However, we suggest that biases introduced from provenance of sample, sequencing protocol, primers and bioinformatics analyses may potentially pose a larger effect on the outcome than sample storage, media and freeze-thaw cycles, also proposed by Lauber et al. [20], and future meta-analyses may confirm this suggestion.

The advantages of using screening samples are large number of sample, population wide selections, well-characterized participants often with colonoscopy data and potentially long follow up times enabling longitudinal design. Some of the available samples and data sources are screening pilots and trials such as NORCCAP and the BCSN trial.

The established association between infections with certain microbes and colorectal cancer [1] can now be studied in depth with new technology and unprecedented accuracy and resolution. There is a need for longitudinal observational studies to define modes of microbial action and confirm a causative relationship for one of the world’s leading causes of cancer death. The results reported here may contribute to expand the use of archived fecal material for this purpose.

## Conclusion

We have shown that it is feasible to use 1) fecal samples used for iFOBT screening and 2) fecal samples frozen for multiple years and potentially thawed several times, in large-scale epidemiological studies on microbiota. The test for fecal occult blood in the iFOBT sample does not introduce cross sample contamination and substantial storage effects, therefore the sample from the collection device may be used in highly sensitive methods such as next generation sequencing*.* The iFOBT samples are comparable in alpha diversity and composition to fresh fecal samples. However, storage and sampling methods have been shown to affect the microbiome composition and diversity, therefore in the case-control design, both cases and controls should be selected from the same sample group and processed in the same way. Care must be taken when using archived fecal samples and optimization of the protocol is critical.

## Additional files

Additional file 1: Distribution of OTU counts in all samples analysed. (PDF 513 kb)

Additional file 2: Table with OTU counts for all samples. (TXT 13318 kb)

Additional file 3: Taxonomical information for all OTUs. (TXT 9091 kb)

Additional file 4: Alpha diversity measurements. (TXT 22 kb)

Additional file 5: Beta diversity measurements. (XLSX 126 kb)

Additional file 6: Differences in microbial composition between iFOBT and archived samples. (PDF 115 kb)

Additional file 7: Statistical differences in microbiota composition between fresh frozen directly and fresh frozen after 48 h in room temperature. (PDF 92 kb)

## Abbreviations

BCSN: The Bowel Cancer Screening in Norway
BSA: Bovine serum albumin
CRC: colorectal cancer
ENC: extraction-negative control
FIT: fecal immunochemical tests (=iFOBT)
FS: flexible sigmoidoscopy
gFOBT: guaiac-based fecal occult blood test
HEPES: 4-(2-hydroxyethyl)-1-piperazineethanesulfonic acid
iFOBT: immunochemical-based fecal occult blood test
NORCCAP: NORwegian Colorectal Cancer Prevention
OTU: Operational taxonomic unit
PBS: phosphate-buffered saline
