# Supplementary figures and images for: Evaluating gut microbiota profiles from archived fecal samples
Source: BMC Gastroenterol. 2018 Nov 8;18:171. doi: 10.1186/s12876-018-0896-6 (PMC6225565; doi:10.1186/s12876-018-0896-6)

Additional File 1:

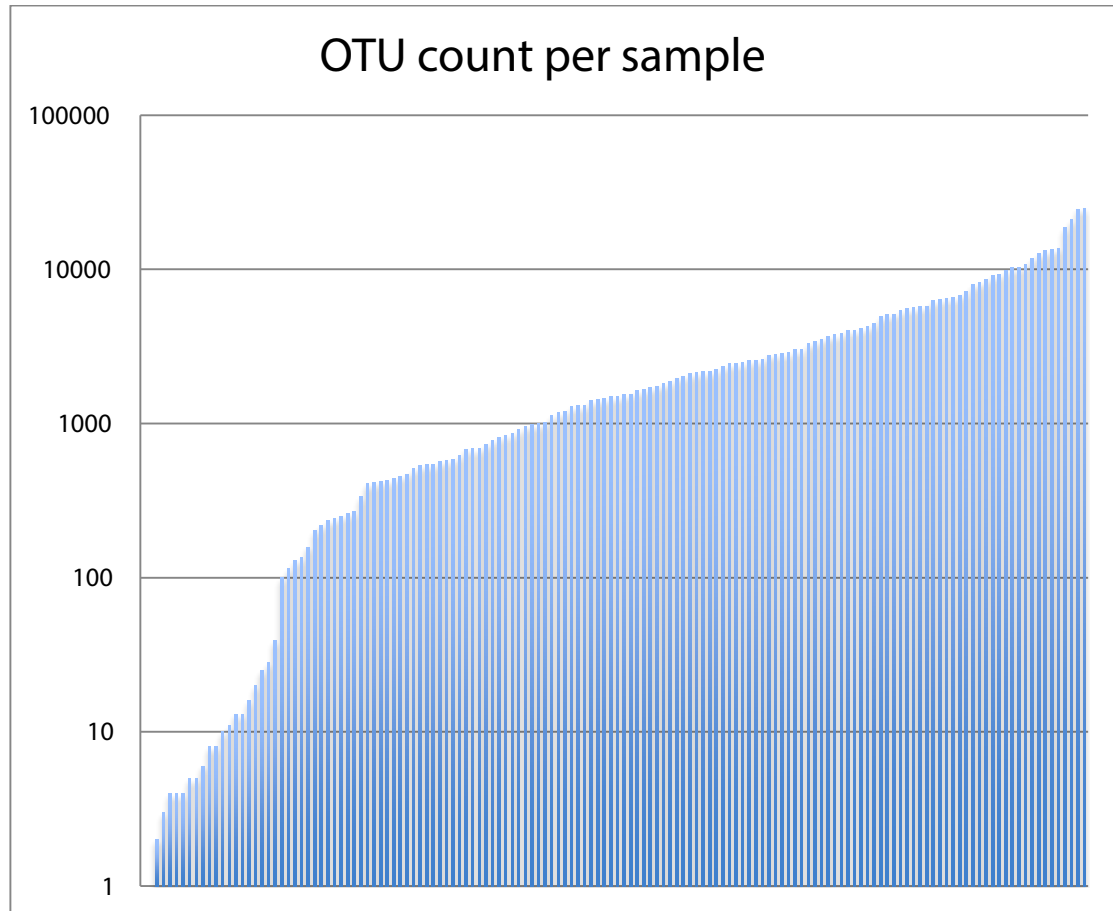

Figure s1: Distribution of OUT counts in all samples analysed

Supplement: Supplementary file 1 — Distribution of OTU counts in all samples analysed. (PDF 513 kb) [file 12876_2018_896_MOESM1_ESM.pdf]
