# Supplementary material for: Evaluating gut microbiota profiles from archived fecal samples
Source: BMC Gastroenterol. 2018 Nov 8;18:171. doi: 10.1186/s12876-018-0896-6 (PMC6225565; doi:10.1186/s12876-018-0896-6)
Supplement: Supplementary file 6 — Differences in microbial composition between iFOBT and archived samples. (PDF 115 kb) [file 12876_2018_896_MOESM6_ESM.pdf]

Table s6: Differences in microbial composition between buffered and archived samples

OTU	logFC	se	pvalues	adjPvalues
Otu04	-1,232	0,201	8,23E-10	2,55E-08
Otu11	1,729	0,296	5,08E-09	7,87E-08
Otu18	1,853	0,370	5,57E-07	5,76E-06
Otu14	-1,526	0,333	4,64E-06	3,59E-05
Otu05	-0,816	0,197	3,31E-05	2,05E-04
Otu09	-0,755	0,273	5,68E-03	2,52E-02
Otu16	0,846	0,306	5,69E-03	2,52E-02
Otu21	0,980	0,372	8,37E-03	3,24E-02
Otu17	-0,869	0,340	1,06E-02	3,67E-02
Otu12	0,638	0,256	1,26E-02	3,92E-02
Otu19	0,730	0,303	1,58E-02	4,45E-02
Otu22	-1,381	0,591	1,94E-02	5,02E-02
Otu28	1,132	0,534	3,41E-02	8,13E-02
Otu32	-1,394	0,747	6,19E-02	1,28E-01
Otu42	1,618	0,858	5,94E-02	1,28E-01
Otu06	-0,357	0,203	7,89E-02	1,53E-01
Otu07	1,442	0,917	1,16E-01	2,11E-01
Otu23	0,858	0,607	1,58E-01	2,71E-01
Otu08	0,353	0,256	1,68E-01	2,74E-01
Otu24	0,337	0,301	2,62E-01	4,06E-01
Otu02	-0,774	0,822	3,46E-01	5,11E-01
Otu29	-0,858	1,047	4,13E-01	5,81E-01
Otu03	-0,531	0,796	5,05E-01	6,26E-01
Otu13	0,171	0,249	4,93E-01	6,26E-01
Otu31	0,553	0,775	4,75E-01	6,26E-01
Otu27	-0,323	0,761	6,71E-01	8,00E-01
Otu43	0,204	0,684	7,65E-01	8,79E-01
Otu01	-0,107	0,713	8,81E-01	9,42E-01
Otu10	-0,038	0,229	8,68E-01	9,42E-01
Otu15	0,032	0,298	9,14E-01	9,45E-01
Otu20	0,015	0,344	9,64E-01	9,64E-01
Otu26	NA	NA	NA	NA
Otu34	NA	NA	NA	NA
Otu36	NA	NA	NA	NA
Otu52	NA	NA	NA	NA
