# Supplementary material for: Evaluating gut microbiota profiles from archived fecal samples
Source: BMC Gastroenterol. 2018 Nov 8;18:171. doi: 10.1186/s12876-018-0896-6 (PMC6225565; doi:10.1186/s12876-018-0896-6)
Supplement: Supplementary file 7 — Statistical differences in microbiota composition between fresh frozen directly and fresh frozen after 48 h in room temperature. (PDF 92 kb) [file 12876_2018_896_MOESM7_ESM.pdf]

Table S7: Statistical differences in microbial composition between fresh frozen directly and fresh frozen after 48h in room temperature

OTU	. logFC	se	pvalues	adjPvalues
Otu32	-0,618	0,647	0,339	0,994
Otu22	-0,504	0,959	0,600	0,994
Otu17	-0,415	0,603	0,492	0,994
Otu05	-0,336	0,623	0,590	0,994
Otu02	-0,307	0,608	0,614	0,994
Otu13	-0,247	0,679	0,716	0,994
Otu12	-0,237	0,848	0,780	0,994
Otu15	-0,203	0,850	0,811	0,994
Otu06	-0,192	0,682	0,778	0,994
Otu09	-0,159	0,934	0,865	0,994
Otu03	-0,152	0,594	0,798	0,994
Otu01	-0,139	0,414	0,736	0,994
Otu04	-0,116	0,958	0,903	0,994
Otu07	-0,102	0,631	0,871	0,994
Otu10	-0,090	0,642	0,889	0,994
Otu08	-0,063	0,898	0,944	0,994
Otu14	-0,009	0,440	0,985	0,994
Otu11	-0,007	0,902	0,994	0,994
Otu19	0,221	0,496	0,656	0,994
Otu18	0,228	0,518	0,659	0,994
Otu24	0,354	0,724	0,625	0,994
Otu21	0,464	0,505	0,358	0,994
Otu16	0,637	0,758	0,401	0,994
Otu28	0,781	0,921	0,396	0,994
Otu20	1,259	0,851	0,139	0,994
